# Using Bi-Seasonal WorldView-2 Multi-Spectral Data and Supervised Random Forest Classification to Map Coastal Plant Communities in Everglades National Park

**DOI:** 10.3390/s18030829

**Published:** 2018-03-09

**Authors:** Kristie S. Wendelberger, Daniel Gann, Jennifer H. Richards

**Affiliations:** 1 The Everglades Foundation, 18001 Old Cutler Road, Palmetto Bay, FL 33157, USA; 2 Department of Biological Sciences, Florida International University, 11200 S.W. 8th Street, Miami, FL 33199, USA; gannd@fiu.edu (D.G.); richards@fiu.edu (J.H.R.); 3 Geographic Information Systems and Remote Sensing Center, Florida International University, 11200 S.W. 8th Street, Miami, FL 33199, USA

**Keywords:** mangrove, species spectral separability, random forest classifier, rare species, habitat monitoring, sea level rise, climate change, conservation

## Abstract

Coastal plant communities are being transformed or lost because of sea level rise (SLR) and land-use change. In conjunction with SLR, the Florida Everglades ecosystem has undergone large-scale drainage and restoration, altering coastal vegetation throughout south Florida. To understand how coastal plant communities are changing over time, accurate mapping techniques are needed that can define plant communities at a fine-enough resolution to detect fine-scale changes. We explored using bi-seasonal versus single-season WorldView-2 satellite data to map three mangrove and four adjacent plant communities, including the buttonwood/glycophyte community that harbors the federally-endangered plant *Chromolaena frustrata*. Bi-seasonal data were more effective than single-season to differentiate all communities of interest. Bi-seasonal data combined with Light Detection and Ranging (LiDAR) elevation data were used to map coastal plant communities of a coastal stretch within Everglades National Park (ENP). Overall map accuracy was 86%. Black and red mangroves were the dominant communities and covered 50% of the study site. All the remaining communities had ≤10% cover, including the buttonwood/glycophyte community. ENP harbors 21 rare coastal species threatened by SLR. The spatially explicit, quantitative data provided by our map provides a fine-scale baseline for monitoring future change in these species’ habitats. Our results also offer a method to monitor vegetation change in other threatened habitats.

## 1. Introduction

Coastal plant communities around the world are being altered or completely lost by sea level rise (SLR) and land-use change [1,2,3]. The global extent of mangroves and associated coastal wetland communities, which are important wildlife and rare species habitats, has shrunken as a result of climate change and anthropogenic disturbance [1,4,5,6], threatening species of concern [4,7]. A plant community migrates as a response to ecological pressures to which it is exposed; therefore, a spatially explicit and quantitative understanding of an ecosystem’s plant community matrix at high spatial and thematic precision is essential for monitoring subtle ecosystem change [4,6] and predicting future species’ habitats. To better understand how mangrove communities are responding to large-scale ecological change, accurate mapping techniques are needed that differentiate both mangrove species community types and adjacent higher-elevation plant communities [4,6].

A number of researchers have had success accurately classifying wetland and mangrove communities using a combination of data sources [4,6,8,9,10,11]. Seasonal differences in phenological stages of various species within a plant community result in variability of spectral signatures that can be utilized to improve classification accuracy [9,12]. Multi-temporal satellite imagery has been shown to accurately classify wetland plant communities over distinct phenological time periods [13,14,15]. In France, multi-spectral, multi-seasonal imagery was combined with Light Detection and Ranging (LiDAR) data to accurately classify wetland vegetation [4,16]. In ecosystems with two distinct seasonal patterns, bi-seasonal imagery can capture these seasonal differences in plant phenology and, therefore, provide spectral signatures that can help differentiate communities that would otherwise be difficult to distinguish remotely [17,18]. 

Using bi-seasonal satellite data to capture differences in plant phenology has been successful in mapping some plant communities [17,18,19]. Because non-native willows (*Salix* spp.) are deciduous during cooler months, reducing shortwave infrared light absorption, Noonan and Chafer [11] satisfactorily used a composite image from bi-seasonal satellite for observation of Earth imagery (SPOT-5) during autumn (complete willow leaf cover) and winter (bare leaf cover) to generate spectral signatures that were distinct from signatures of the adjacent native, evergreen, and riparian vegetation. South Florida’s Everglades, the region for this study, has two distinct seasons, wet (June–October) and dry (November–May) [20]. Bi-seasonal satellite imagery was effective in discriminating plant communities in the freshwater Everglades [18] but has not been evaluated for the coastal fringe. Everglades coastal communities undergo drought and salt stress during the dry season as compared to the wet season [7,21]. Plant communities display drought and salinity stress through changes in their leaf physiology [22,23] that then affect the spectral signature they produce [24]. South Florida coastal forests, including buttonwood forests, exhibit seasonality in litterfall, which tracks seasonality in leaf production, generally displaying a high wet/low dry pattern in litterfall biomass [25]. These seasonal phenological patterns documented in the field suggest that the use of bi-seasonal data for remotely-sensed classification of the Everglades coastal fringe may be a viable option.

Florida’s southern coast in Everglades National Park (ENP), FL, USA, has three mangrove and four coastal plant community types that are distributed across the landscape as a response to elevation, salinity, and inundation time [7,26,27,28]. The Buttonwood Embankment, which harbors these communities, is an approximately 60 × 1 km^2^ stretch of elevated land averaging 45 cm above sea level along the southern tip of Florida [29,30]. The Embankment provides habitat for the entire mainland range of the federally endangered plant *Chromolaena frustrata* (B.L.Rob.) R.M.King & H.Rob., as well as several state endangered plant species [7]. Historically, freshwater flowed from the north toward saline Florida Bay, forming fresh waterbodies to the north of the embankment [27,31]. Because of the low elevation gradients in the area, small increases in sea level or decreases in freshwater head can have large impacts on plant communities and, therefore, species of concern [7]. Today, after 100 years of SLR and anthropogenic drying of the Everglades ecosystem, the waterbodies to the north of the embankment are now brackish to marine [27]. 

Various habitat types in the southern coastal Everglades harbor different rare plant species [7] and small changes in elevation influence species composition [28,32,33], thus detecting subtle changes in plant composition at high spatial precision along the mangrove fringe is important for rare species habitat monitoring. There are two buttonwood forest communities distinguished by different understory vegetation in our study area; one harbors glycophyte species, including *C. frustrata*, while the other is slightly lower in elevation, dominated by halophytic species, and does not have *C. frustrata*. Our overall objective, in this study, was to create a detailed map of the vegetation communities in our study area, including distinguishing *C. frustrata* habitat. With a base map, we can produce future maps of the site in order to monitor change that may impact the rare species. Because this area has not been mapped at the spatial resolution necessary for such monitoring, our research objective was to assess whether using bi-seasonal, multi-spectral satellite data with high spatial resolution would be more effective in separating plant communities than would data from a single season. We also evaluated which community types benefited most from bi- versus single-season data. 

Model-based accuracies showed that the combination of data sources was successful in distinguishing the three mangrove species and the other four coastal plant communities in the study area with an overall accuracy of 86%. We showed that bi-seasonal data more accurately distinguished plant community types than either single season alone. Additionally, we were able to distinguish buttonwood/glycophyte forest—the habitat harboring the federally endangered *C. frustrata*—from adjacent buttonwood/halophyte forest at a greater accuracy than when using single-season data. Black and red mangroves had the greatest percent cover, while the highest elevation plant community—tropical hardwood hammock—had the least percent cover. The percent cover for each of the community types provides an understanding of the current plant community matrix in the area mapped. These data, which are both spatially explicit and quantitative, provide a baseline to monitor future changes in mangrove and other coastal plant communities.

## 2. Materials and Methods

### 2.1. Study Area

**Figure 1:**
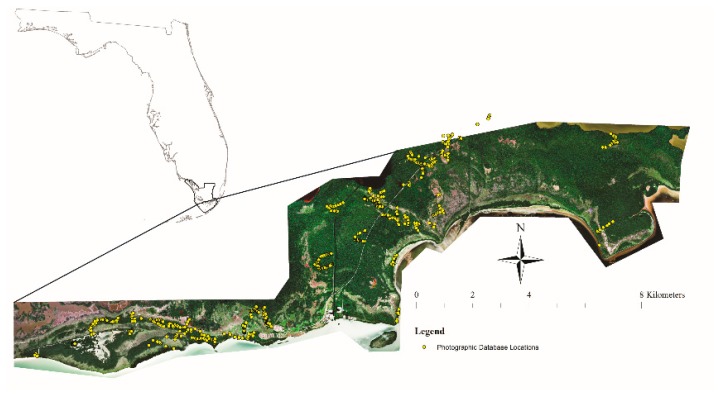
DigitalGlobe WorldView-2 satellite image (red, green, blue bands displayed in RGB) of 71 km^2^ study site along the south Florida coast around Flamingo, Everglades National Park (ENP) with Florida Bay to the south. The inset shows Florida with ENP outlined and location of the study area indicated. The ENP road to Flamingo is visible, running NE to SW through the center of the image. Mapped yellow points are the GPS points associated with the 2012–2015 photographic database of the study area.

The study area was a 71 km^2^ strip along the coast of Everglades National Park (ENP) (25°19′0″ N, 80°56′0″ W) in southern Florida, U.S.A. (Figure 1). Southern Florida is humid and subtropical with a distinct warm (mean 25 °C) wet season from June to October and cool (mean 16 °C) dry season from November to May [20]. Average annual rainfall is between 1000 to 1630 mm with more than half the rain falling between June and September and often coming from hurricanes and tropical storms. April and May are usually the driest months [20,34]. 

### 2.2. Community Type Descriptions

In the field, we identified seven plant communities that were recognized frequently at a spatial scale of 4 m^2^, the scale at which we were interested in mapping these communities. The seven plant communities were:

(1) *Black Mangrove Forest:* This forest is dominated by black mangrove (*Avicennia germinans* (L.)) with few associated woody species [35], except for occasional white mangrove (*Laguncularia racemosa* (L.) C.F.Gaertn) or red mangrove (*Rhizophora mangle* L.) found in either the canopy or understory. At times, areas of young black mangrove forest have halophyte species in the understory. Black mangrove forests are considered the most salt-tolerant of the three mangroves found in south Florida [36].
(2) *Red Mangrove Forest:* This forest is dominated by *Rhizophora mangle* in the canopy and has little to no understory vegetation [35]. Occasional *A. germinans* are found scattered throughout; *L. racemosa* is found even less commonly. Red mangrove forests are considered the most inundation-tolerant of the three mangrove types and are less salt-tolerant than black mangroves [36].
(3) *White Mangrove Forest:* This forest is dominated by *Laguncularia racemosa* in the canopy; often halophytes such as *Batis maritima* L., *Sarcocornia perennis* (Mill.) A.J. Scott, and *Suaeda linearis* (Elliott) Moq. are found in the understory. This community is most often found in irregularly flooded areas [35] and is the least salt- and inundation-tolerant of the three mangrove species found in south Florida [36].
(4) *Buttonwood/Glycophyte Forest:* Buttonwood (*Conocarpus erectus* L.) is the dominant canopy species of buttonwood forests, but other woody species and a diverse herbaceous understory are also found in this community [37]. Temperature, salinity, tidal fluctuation, substrate, and wave energy influence the size and extent of buttonwood forests [38], which often grade into salt marsh, coastal berm, rockland hammock, coastal hardwood hammock, and coastal rock barren [38]. This community sustains freshwater flooding during the wet season and is dry during the dry season [38]. Buttonwood forests (mean elevation 29 ± 3 cm) maintain an average groundwater table of −33 ± 1 cm and (26–29.5) ± 0.4‰ groundwater salinity [7,37].
(5) *Buttonwood/Halophyte Forest: C. erectus* is the only canopy tree species in buttonwood/halophyte forests. The understory is comprised of halophytic species such as *Batis maritima, Borrichia frutescens* (L.) DC., *Distichlis spicata* (L.) Greene, *Sarcocornia perennis*, and *Suaeda linearis* [37]. Buttonwood/halophyte forest (also called buttonwood prairies [37]) (mean elevation 18 ± 3 cm) show a mean groundwater table at −32 ± 2 cm and average groundwater table salinity of 38.8 ± 0.6‰ [37].
(6) *Halophyte Prairie:* These prairies are comprised of *Batis maritima, Borrechia frutescens*, *D. spicata, Sarcocornia perennis,* and *Sueda linearis*, as well as other less common species; there is no canopy species [37]. Halophyte prairies have marl soils and slightly higher elevation than adjacent black and white mangrove forests [33]. In halophyte prairies, standing water that is brackish to freshwater is present for months during the wet season. Evaporation and lack of drainage cause these communities to become hypersaline during the dry season [32].
(7) *Coastal Tropical Hardwood Hammocks:* Coastal tropical hardwood hammocks are biodiverse. This community includes a variety of tropical tree and shrub species [7,35] and a different suite of herbaceous species than are found in the mangrove communities [7]. Ground cover is often limited in closed canopy areas and abundant in areas where canopy disturbance has occurred or where this community intergrades with buttonwood forest [7]. Coastal tropical hardwood hammocks are the least salt-tolerant of all the coastal community types and reside at the highest elevation (mean elevation 29 ± 3 cm) [37].

Two invasive species, *Schinus terebinthifolius* Raddi (Brazilian pepper) and *Colubrina asiatica* (L.) Brongn (latherleaf), were prominent in the area and found across several forest types, causing confusion during the classification process; we created a classification category for each of these species to improve accuracy in the other community types. We also included a class for mud flats, which were open areas of bare soil or areas covered with periphyton mats, and a class for open water.

### 2.3. Classifier Evaluation and Community Prediction for the Study Area

Creating the Classification Training Set: For each of the seven plant community types plus mud flats and open water, a set of field GPS points were acquired using a Garmin 60C× (Garmin International, Inc., Olathe, KS, USA) with a 1–7 m accuracy. To account for this accuracy in relation to our scale of interest, we collected the points more than 7 m from the edge of the community in plant community patches that were greater than 14 m in diameter. In addition to field training points, we digitized training samples using a digital stereoplotter (DAT/EM Systems International, Anchorage, AK, USA) with 2009 aerial photography and the ArcMap 10.3.1 (ESRI 2014) basemap. A total of 17,166 training points were used in the classification. The number of points per vegetation class varied from 557 (buttonwood/glycophyte community) through 4408 (halophyte prairie community). The number of points varied because classes that had more variability in their spectral signatures needed more training points, and some communities, such as the buttonwood/glycophyte community, were rare. During the ground survey, we documented community types at the GPS points with pictures in each cardinal direction, plus zenith and nadir, creating a 2012–2015 photographic vegetation database (4356 pictures at 730 points, Figure 1). This database provided temporal and spatial photographic documentation of the study site, which was used to select the additional digitized training points.

Satellite Data and Image Processing: We used WorldView-2 (WV2) satellite data acquired at the end of the wet (11 December 2011) and dry (13 April 2013) seasons. The WV2 imagery has 2 × 2 m^2^ resolution and eight spectral bands (coastal: 400–450 nm; blue: 450–510 nm; green: 510–580 nm; yellow: 585–625 nm; red: 630–690 nm; red edge: 705–745 nm; near-IR1: 770–895 nm; and near-IR2: 860–1040 nm). We chose images from the end of the wet and dry seasons because species are at their maximum response level to both seasons at those times. There was no major storm event between December 2011 and 2013 that affected the vegetation in the area. Therefore, the different years’ wet and dry seasons were assumed comparable. Both images were geometrically corrected using the orthorectification module in ENVI version 5.2 (Exelis Visual Information Solutions, Boulder, CO, USA). Root mean square errors for the December 2011 and the April 2013 images for the 2009 stereo aerial photography reference data sets were 5.9 m and 5.0 m, respectively, and in reference to the ArcGIS basemap, they were 4.5 m and 4.8 m, respectively.

Images were atmospherically corrected using the Fast Line-of-sight Atmospheric Analysis of Hypercubes (FLAASH) module in ENVI version 5.2 (Exelis Visual Information Solutions, Boulder, CO, USA). The selected atmospheric model was “Tropical”, and the aerosol model was “Maritime” for both images, with the initial visibility parameter set to 80 km for the April 2013 image and 40 km for the December 2011 image.

For each band of the two images, we generated local texture variables using a 3 × 3 kernel. The local texture variables we calculated for the kernel were reflectance mean, range, and standard deviation. In addition, we calculated the Normalized Difference Vegetation Index (NDVI) for each image. Since elevation differences were expected to provide valuable information for plant community presence, a digital terrain model (DTM) was included. The LiDAR-derived DTM, acquired in 2007–2008 and gridded at 5ft spatial resolution by the Florida Division of Emergency Management, had a vertical accuracy of 0.18 m (CI = 95%) [39]; to match the resolution of WV2, the DTM was resampled to 2 m resolution using nearest-neighbor interpolation. 

Feature Set Evaluation: To assess model-based overall accuracy differences between single wet- or dry-season data and between each single season and the combined bi-seasonal data, we constructed three feature sets for optimal feature selection using the wrapper method [40]. The three feature sets were (1) bi-seasonal data with 33 features each plus the DTM, totaling 67 features; (2) single wet; and (3) single dry season data with 33 features plus DTM each (Table 1). The classifier we chose was the random forest [41], which has been applied successfully in many remote sensing applications [42,43].

For each training sample, variables for the three feature sets were extracted. Within each feature set, secondary feature selection was performed and feature importance was assessed using built-in bootstrapping and cross-validation procedures inherent to the random forest algorithm as implemented in the R package caret [44]. The parameters used in the random forest procedures as implemented in the caret package were the number of trees, the number of randomly selected features at each node, and folds for the cross-validation procedure. The number of trees was consistently set to 1000. Parameter tuning was employed to determine the best number of features to be considered and randomly selected at each node (“mtry”). This parameter was tuned for the range of 2 features up to the number of features available in each feature set (e.g., for a single-season feature set with 34 features, “mtry” ranged from 2 to 34 and for the bi-seasonal set, from 2 to 67) with the evaluation criterion set to “accuracy”. The number of folds for cross-validation was set to 10.

Classifier performance for the three sets was assessed with the overall, model-based, out-of-bag (oob) error estimates obtained with a 10-fold cross-validation [45]. We evaluated which communities benefited most from bi- versus single-season data and which features were most effective within each feature set. The criterion for feature importance was decrease in mean accuracy, which was provided by the output of the random forest algorithm.

Design-Based Map Accuracy: The final map was predicted from the feature set that provided the highest model-based overall accuracy. For those areas in the satellite data that were cloud covered, we masked by manually digitizing cloud and cloud shadow masks. For the cloud-masked areas only the satellite image that did not contain clouds and the DTM were used to predict the final class label. The amount of cloud and cloud shadow were 0.9% for the dry and 0.6% for the wet season images. For the final map, a minimum mapping unit of 20 m^2^ was chosen. Pixel clumps for each community that were below the 20 m^2^ threshold (nine-pixel neighborhood) were reclassified using a majority rule for a 3 × 3 moving window; clump gaps were filled iteratively from the outside in until all pixels were assigned a class label. Map accuracy of the final map was evaluated with a design-based, stratified, random-sampling method where the number of samples was calculated based on a multinomial distribution evaluating an 85% accuracy at a 95% confidence [46,47]. Stratifying by community type, 53 pixels per community were randomly selected for evaluation. Because the two invasive species, *S. terebinthifolius* and *C. asiatica,* had the smallest area and were difficult to distinguish from aerial photography, we combined those two categories for the accuracy assessment. We used a digital stereoplotter (DAT/EM Systems International, Anchorage, AK, USA) with 2009 aerial photography and the ArcMap 10.3.1 (ESRI 2014) basemap to label each of the randomly-sampled reference pixels without prior knowledge of the model classification. Those pixels that we could not identify in either of the aerial photography sources were verified in the field. 

To quantify accuracy of the map and estimate area of each community type across the 71 km^2^ area, we evaluated the error matrix of the map label versus reference pixel labels. Using the reference data of the random samples and mapped class proportions, the areal cover estimates for each class were adjusted to eliminate bias that can be attributed to classification errors [48,49]. Confidence intervals for the error-adjusted area estimates were calculated to quantify the sampling variability of the estimated area using the procedures recommended by Olofsson et al. [48] and Stehman [49]. Mapped proportions of each class and estimated error-corrected areal estimates were then used to adjust overall, user’s and producer’s accuracies with standard errors and 95% confidence intervals [48]. All data processing and analysis was performed in R [50] using packages raster [51], rgdal [52], and caret [44]. 

## 3. Results

### 3.1. Bi-Seasonal Versus Single Season Signature Assessment

Distinguishing the seven plant communities in our study area, model-based accuracy was highest for the bi-seasonal feature dataset when compared to wet- or dry-season alone (Table 2). The bi-seasonal dataset also showed lower class-specific omission errors across all community types than either the wet- or dry-season data (Table 2). 

The feature that had the largest mean decrease in accuracy when dropped from the feature set differed among models. For the bi-seasonal and wet-season models, NDVI had the greatest mean decrease in accuracy (15% and 18%, respectively), while for the dry season the DTM contributed most to the overall accuracy (mean decrease in accuracy = 16%). For the dry season model, NDVI was only the 15th most important variable out of 34 total. The DTM ranked second in importance for the bi-seasonal and wet season models, while the reflectance mean for Short-Wave Infared-2 (SWIR) (band 8) was second for the dry season. The feature set that provided the highest separability between the two buttonwood communities was the wet season data (Table 2). For the dry and the bi-seasonal data set, the 3 × 3 mean SWIR-1 (band 7) of the dry season image was the most important spectral information in detecting the buttonwood-glycophyte class, and for the wet season, it was the mean of SWIR-2 (band 8) in detecting buttonwood/halophyte.

### 3.2. Vegetation Map 

Map Design-Based Accuracy Assessment: overall adjusted design-based map accuracy was 86%. Looking at adjusted user’s accuracy, buttonwood/halophyte, buttonwood/glycophyte, and white mangrove forests had the highest accuracies (Table 3) followed by red and black mangrove forests (Table 3). Halophyte prairies and tropical hardwood hammock had the lowest adjusted user’s accuracies (Table 3). 

### 3.3. Community Area and Percent Cover

Evaluating the sources of confusion among classified communities, mangroves, and upland communities were confused both within and between the community types. The largest proportion of misclassified black mangrove forest reference points were classified as buttonwood/glycophyte forest, followed by red mangrove, then white mangrove and buttonwood/halophyte (Table 4). Buttonwood/glycophyte forest was confused most frequently with black mangrove forest and buttonwood/halophyte forest (Table 4). Buttonwood/halophyte forest was most often mis-classified as buttonwood/glycophyte forest, then halophyte prairie (Table 4). Hardwood hammock was most frequently mis-classified as buttonwood/halophyte forest, followed by black mangrove. Red mangrove forest was confused most commonly with black mangrove, then hardwood hammock and white mangrove (Table 4). White mangroves were most often confused with buttonwood/halophyte, then buttonwood/glycophyte (Table 4). 

**Figure 2:**
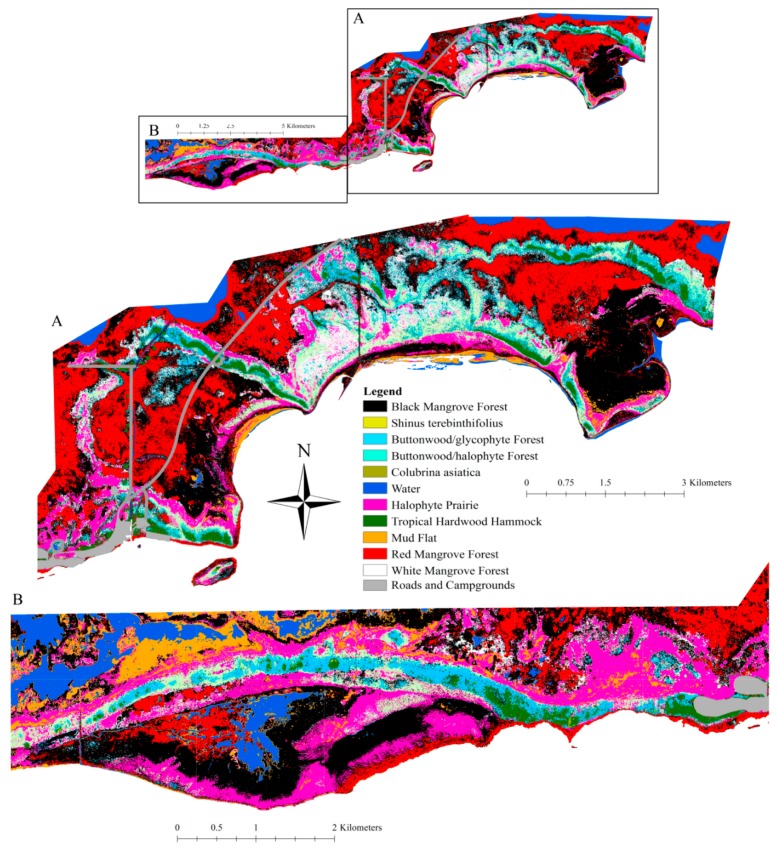
The 20 m^2^ resolution vegetation map of the 71 km^2^ study area, Flamingo, Everglades National Park. The overview shows the entire 71 km^2^ area. (**A**) The enlarged eastern portion of the map; (**B**) the enlarged western portion.

Individual plant communities covered from 183 ha to 2015 ha in the 71 km^2^ study area (Table 3). Black mangrove forest occupied the greatest area and percent cover (Table 3; Figure 2). Red mangrove forest was second most dominant (Table 3; Figure 2), followed by halophyte prairie (Table 3; Figure 2). The other community types had ≤10% cover each (Table 3; Figure 2). Tropical hardwood hammock was the least common vegetation type (3% cover; Table 3; Figure 2). The two invasive species, *S. terebinthifolius* and *C. asiatica*, had 3% cover when combined (Table 3; Figure 2). *Schinus terebinthifolius* tended to be found in tropical hardwood hammock and red mangrove forest (K. Wendelberger, personal observations).

**Figure 3:**
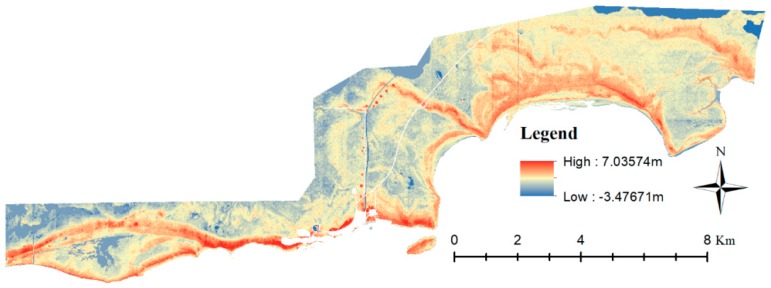
Digital Terrain Model (DTM) of the 71 km^2^ study site, Flamingo, Everglades National Park. Areas with highest elevations are red; areas with lowest elevations are dark blue. The higher elevation buttonwood embankment is seen as the highest elevation natural area throughout the study site. The circles of high elevation along the Buttonwood Canal (center of image) are likely spoil piles from when the canal was built. The roads and campgrounds are white. The DTM was created with LiDAR data flown by the Florida Division of Emergency Management in 2007–2008.

The DTM showed patterns of higher elevation areas interwoven with lower elevation areas throughout the 71 km^2^ study site (Figure 3). Tropical hardwood hammock, buttonwood/glycophyte and buttonwood/halophyte forests tended to follow a sequential pattern along the elevation gradient (Figure 2 and Figure 3). Tropical hardwood hammocks were found at the highest elevation; buttonwood/glycophyte forests were found on slightly lower elevation on either side of tropical hardwood hammocks; and buttonwood/halophyte forest occupied the lower sides of buttonwood/glycophyte forests (Figure 2 and Figure 3). Halophyte prairie was typically located in low spots adjacent to buttonwood/halophyte forests. Black and red mangroves were found in the lowest elevations in the area (Figure 2 and Figure 3). 

## 4. Discussion and Conclusions

Recent changes along southern Florida’s coast have made the development of rapid, efficient, and accurate mapping techniques more urgent. In the past century, a reduction of freshwater flow and increasing rates of SLR have substantially increased the rate of change found along Florida’s coast [7,19,27,28,30,53,54,55,56,57]. For example, Ross et al. [28] found that between 1952 and 2000, graminoid marshes in the eastern portion of the southern Everglades transitioned into mangrove scrub. In addition to drying, there has been more than a 23 cm increase in sea level along south Florida’s coast in the last century [58], and sea levels are expected to continue to rise at a faster rate [59]. A diverse coastal plant community matrix is critical to the health of both human and natural communities [36,60,61,62,63,64], and coastal communities are considered to be a hotspot for high rates of CO_2_ sequestration [63,64]. Understanding how plant communities are distributed across the plant community matrix is essential to knowing what kind of conservation actions are needed in the face of such large-scale change.

Our analysis of single- versus bi-seasonal satellite data demonstrated an effective mapping technique for Florida’s mangrove and coastal communities, which provided a fine-scale baseline map for monitoring future change. Using bi-seasonal WV2 satellite imagery—classifying communities based on the information provided by the spectral bands from both the wet and dry season data—was more effective than using single-season data for distinguishing Florida’s coastal communities. Bi- and multi-seasonal satellite data have been shown to be effective when classifying plant communities in other regions [11,12,16,18]. Such data can also distinguish among mangrove physiological states, e.g., Wang and Sousa [24] found good spectral discrimination between mangroves under nutrient and drought stress as compared to mangroves under non-stressed conditions. During south Florida’s dry season, coastal plant species are subjected to both drier and more saline conditions as compared to the wet season. These coastal communities have been shown to exhibit wet/dry seasonality in biomass production in southern Florida as well as other locations, although the specific pattern of seasonality can vary with location, species, and/or environment (especially temperature, hydrology, and salinity) [25,65,66,67,68]. 

The utility of bi-seasonal remotely-sensed data in fine-scale mapping of coastal communities probably relies on species’ variations in seasonality, which translate into different physiological states, to distinguish among coastal communities. The NDVI was the most important variable for classifying the wet- and bi-seasonal data. The ability of NDVI to help distinguish among species suggests that the different species have different phenological patterns in response to the wet/dry seasonality. The NDVI reflects the general greenness of vegetation and could be expected to differ between wet and dry seasons even in the sub-tropical coastal plant communities of south Florida. In our study, the variable most important in differentiating communities differed between wet and dry season images, so classification using both benefited from the diverse data supplied by the two images. For example, we were able to differentiate between two communities with similar overstory vegetation. Higher elevation buttonwood/glycophyte and lower elevation buttonwood/halophyte forests have similar overstory vegetation, are commonly spatially adjacent to each other on the landscape, and are found on land with only a few centimeters difference in elevation. The bi-seasonal data, however, successfully discriminated between the two communities, which was mainly attributable to the seasonal reflectance differences in the SWIR wavelengths. 

Our map shows that the ENP coastal communities are still quite diverse, maintaining a complex matrix of black, red, and white mangrove forests, halophyte prairie, two buttonwood communities (glycophyte and halophyte), and tropical hardwood hammock. The Buttonwood Embankment [26,27], where the upland communities grow, is a higher elevation strip along Florida’s southern coast. To the north of the embankment, there is over 8km of lower elevation land before elevations return to levels like the embankment’s highest locations. Therefore, an inland migration of coastal tropical hardwood hammock and buttonwood forests in response to SLR is unlikely to occur through the low-lying areas. These upland coastal forests in ENP maintain 21 rare plant species that are threatened by SLR, including *C. frustrata* [7]. It is expected that continued sea level rise and anthropogenic drying will result in buttonwood/glycophyte forest transitioning into buttonwood/halophyte forest, which does not support the federally endangered *C. frustrata.* Coastal tropical hardwood hammock is the highest elevation community along the Embankment and has high plant diversity, but its position at the top makes it more vulnerable to the effects of SLR than the surrounding communities. Similar to the threat of extinction that global warming poses to alpine communities in tropical mountains [69], these forests are at risk of being pushed to extinction, but along a much more subtle elevation gradient and driven by changes in salinity and hydrology rather than temperature. 

This research has produced two important results: (1) a mapping technique that can detect and quantify south Florida coastal plant communities at a fine spatial scale with acceptable accuracy; and (2) a baseline map that can be compared to future maps to quantify loss of rare plant habitats and of coastal tropical hardwood hammock. The coupled stresses of anthropogenic landscape modifications and SLR are the driving factors in south Florida’s coastal wetland loss [3]. Creating a healthy Everglades ecosystem through increased freshwater flow would eliminate or mediate stresses from anthropogenic modifications. Therefore, restoring the hydrologic regime of the Everglades ecosystem is critical to maintaining a coastal community matrix that is as resilient to SLR as possible. The objective of the Comprehensive Everglades Restoration Plan is to increase freshwater flow into ENP. A stronger freshwater head would push back coastal saltwater intrusion, allowing a freshwater lens to form in areas where it no longer exists or is thinning [60]. This would create an environment where the highest elevation communities that harbor the rarest plant species would either be able to expand or at least to maintain their current area, giving species and people time to respond to the press of SLR. Bi-seasonal WV2 maps of the coastal areas made over time can track whether restoration efforts are successful in maintaining a healthy coastal plant community. In addition, the methods presented here provide techniques that can help monitor vegetation change in other threatened habitats.

## Figures and Tables

**Table 1:** Features used in single- and bi-seasonal classifier evaluation.

	December 2011	April 2013	Bi-Seasonal Total
Reflectance Values	8 (1/band)	8 (1/band)	16
Mean	8 (1/band)	8 (1/band)	16
Range	8 (1/band)	8 (1/band)	16
Std Dev	8 (1/band)	8 (1/band)	16
NDVI	1	1	2
DTM	1	1	1
Total	34	34	67

**Table 2:** WorldView-2 random forest classifier models evaluated for the wet season, dry season, and both the wet and dry seasons combined (bi-seasonal) to determine which model would produce the most accurate classification for each community type. Numbers are the percent omission error for each class within each classified model. Columns Wet-Dry, Bi-Wet, and Bi-Dry give the difference in percent error between models for each community; the smaller the absolute value of the number, the more similar those models classified that community type. If a number is positive, the second model in the pair was better (smaller error), while if a number is negative, the first model was better. Overall percent accuracy and standard deviation for each model are given at the bottom. Community types are described in the text: Buttonwood/glyco = Buttonwood/glycophyte; Buttonwood/halo = Buttonwood/halophyte; Hardwood hamm = Hardwood hammock.

	Community	Wet Season	Dry Season	Bi-Seasonal	Wet-Dry	Bi-Wet	Bi-Dry
**Class Specific Error**	Black mangrove	16.5	9.3	8.4	7.2	−8.1	−0.9
	Buttonwood/glyco	63.6	38.7	35.7	24.9	−27.9	−3.0
	Buttonwood/halo	55.5	37.9	34.1	17.6	−21.4	−3.8
	Halophyte prairie	6.8	6.7	4.6	0.1	−2.2	−2.1
	Hardwood hamm	10.8	7.3	6.1	3.5	−4.7	−1.2
	Red mangrove	18.8	16.2	13.8	2.6	−5.0	−2.4
	White mangrove	37.1	27.9	25.5	9.2	−11.6	−2.4
	Mud flat	12.4	22.6	10.7	−10.2	−1.7	−11.9
	Water	0.0	0.1	0.1	−0.1	0.1	0.0
	Overall accuracy	80.1 ± 1.1	84.2 ± 0.9	87.2 ± 1.0	−4.1%	7.1%	3.0%

**Table 3:** Original and adjusted area and accuracy of classified community types in the 71 km^2^ study area, Flamingo, Everglades National Park, Florida. Columns are original mapped area (ha), proportion of area covered (ha), adjusted area (ha), adjusted area %cover, unadjusted community area bias proportion, proportion adjusted user’s and producer’s accuracies, standard errors, and upper and lower 95% confidence intervals. Because it was difficult to distinguish pixels of the two invasive species, *Schinus terebinthifolius* and *Colubrina asiatica*, and their area was small, we combined those two categories (called Invasive species in the table) for the accuracy assessment. Community types are described in the text: Buttonwood/glyco = Buttonwood/glycophyte; Buttonwood/halo = Buttonwood/halophyte; Hardwood hamm = Hardwood hammock.

Community	Area (ha)	Proportional Area (ha)	Adj. Area (ha)	% Cover of Adj. Area	Adj. Area Std. Error	Adj. Area Lower 95% CI	Adj. Area Upper 95% CI	Proportional Area Bias	Proportion Adj. User’s Accuracy	Adj. User’s Accuracy Std. Error	Adj. User’s Accuracy Lower 95% CI	Adj. User’s Accuracy Upper 95% CI	Proportion Adj. Producer’s Accuracy	Adj. Producer’s Accuracy Std. Error	Adj. Producer’s Accuracy Lower 95% CI	Adj. Producer’s Accuracy Upper 95% CI
Black mangrove	2158.5	0.31	2014.5	28.5	63.3	1890.4	2138.6	0.85	0.85	0.05	0.75	0.95	0.91	0.03	0.85	0.97
Invasive species	16.4	0.00	227.4	3.2	41.6	145.8	309.0	0.89	0.91	0.04	0.84	0.99	0.07	0.04	−0.02	0.15
Buttonwood/glyco	429.8	0.06	615.2	8.7	32.6	551.4	679.1	0.91	0.91	0.04	0.83	0.99	0.63	0.07	0.51	0.76
Buttonwood/halo	653.2	0.09	667.5	9.5	4.1	659.4	675.6	1.00	1.00	0.00	1.00	1.00	0.98	0.01	0.96	1.00
Halophyte prairie	899.5	0.13	703.5	9.9	31.8	641.2	765.8	0.74	0.74	0.06	0.62	0.86	0.94	0.04	0.86	1.02
Hardwood hamm	239.2	0.03	182.9	2.6	14.6	154.2	211.6	0.62	0.62	0.07	0.49	0.76	0.82	0.11	0.61	1.02
Red mangrove	1493.0	0.21	1470.6	20.8	46.3	1379.8	1561.4	0.89	0.89	0.05	0.80	0.97	0.90	0.04	0.82	0.98
White mangrove	511.6	0.07	532.6	7.5	27.4	478.9	586.3	0.91	0.91	0.04	0.83	0.99	0.87	0.08	0.71	1.03
Mud flat	253.7	0.04	240.9	3.4	7.9	225.4	256.4	0.89	0.89	0.05	0.80	0.97	0.94	0.04	0.85	1.02
Water	408.6	0.06	409.7	5.8	14.5	381.3	438.1	0.89	0.89	0.05	0.80	0.97	0.89	0.05	0.79	0.98
Total	7063.5	1.00	7064.8	100	Adjusted accuracy			86.02%								

**Table 4:** Stratified-random probability accuracy assessment error matrix, stratifying by community types for the 71 km^2^ study area, Flamingo, Everglades National Park, Florida. Rows are classification-derived map labels; columns are the reference labels. The main diagonal (in bold) shows the proportion of correctly classified pixels, the user’s accuracy. Because it was difficult to distinguish pixels of the two invasive species, *Schinus terebinthifolius* and *Colubrina asiatica*, and their area was small, those categories were combined for the accuracy assessment. Community types described in the text: Buttonwood/glyco = Buttonwood/glycophyte; Buttonwood/halo = Buttonwood/halophyte.

	Black Mangrove	Buttonwood/gLyco	Buttonwood/hAlo	Water	Halophyte Prairie	Hardwood Hammock	Invasive Species	Mud Flat	Red Mangrove	White Mangrove
Black mangrove	**0.85**	0.08	0.02	0.00	0.00	0.00	0.00	0.00	0.04	0.02
Buttonwood/glyco	0.06	**0.89**	0.06	0.00	0.00	0.00	0.00	0.00	0.00	0.00
Buttonwood/halo	0.00	0.06	**0.91**	0.00	0.04	0.00	0.00	0.00	0.00	0.00
Water	0.00	0.00	0.00	**1.00**	0.00	0.00	0.00	0.00	0.00	0.00
Halophyte prairie	0.02	0.04	0.02	0.00	**0.89**	0.00	0.00	0.04	0.00	0.00
Hardwood hammock	0.06	0.00	0.11	0.00	0.02	**0.74**	0.04	0.00	0.04	0.00
Invasive species	0.02	0.00	0.15	0.00	0.02	0.06	**0.62**	0.00	0.13	0.00
Mud flat	0.02	0.00	0.00	0.06	0.04	0.00	0.00	**0.89**	0.00	0.00
Red mangrove	0.08	0.00	0.00	0.00	0.00	0.02	0.00	0.00	**0.89**	0.02
White mangrove	0.00	0.02	0.08	0.00	0.00	0.00	0.00	0.00	0.00	**0.91**
Accuracy: 85.66%	95% CI: (0.8238, 0.8853)									
